# Synthesis of a New Group of Aliphatic Hydrazide Derivatives and the Correlations between Their Molecular Structure and Biological Activity

**DOI:** 10.3390/molecules17033560

**Published:** 2012-03-22

**Authors:** Małgorzata Kostecka

**Keywords:** hydrazides, *Fusarium solani*, *Fusarium oxysporum*, antifungal activity, synthesis

## Abstract

In view of the growing demand for new compounds showing biological activity against pathogenic microorganisms, such as pathogenic and phytopathogenic fungi, the objective of this study was to synthesize a new group of aliphatic and aromatic derivatives of hydrazide. In consequence of the reactions observed during synthesis, the resulting compounds retained their linear structure. Their structure and lipophilicity, measured by high-performance liquid chromatography (HPLC), were analyzed. Correlations were determined between the compounds’ molecular parameters and biological activity against *Fusarium solani* and *Fusarium oxysporum* fungi. The investigated compounds were also examined for their antifungal activity against *Aspergillus fumigatus*. The obtained results indicate that compounds with fluorine-containing substituents penetrate the cell structure more effectively and are characterized by higher antifungal potential than analogues with different substituents.

## 1. Introduction

Heterocyclic compounds, mainly those containing sulfur and nitrogen atoms, are an interesting topic of research in planning organic synthesis due to their biological properties [1,2]. The C(=S)-NH- group [3,4] and the -NH-NH- hydrazide fragment [5,6,7] play a very important role owing to their potentially high antifungal, antibacterial [8,9], antiviral (HIV-1) [10] and anti-malarial activity. Recent research has demonstrated the anticarcinogenic potential of modified hydrazide derivatives [11,12,13], and *in vitro* and *in vivo* experiments have shown that when administered in very small doses, the discussed compounds can induce the death of neoplastic cells without harming healthy cells [14]. Hydrazide derivatives can be applied in crop protection products, in particular those designed to fight phytopathogenic fungi such as *Fusarium solani* and *Fusarium culmorum* which cause head blight and contaminate crops with mycotoxins—Toxic metabolites of their biological activity [15]. Those products could replace conventional fungicides to which most plant pathogens have developed a resistance. For this reason, the synthesized hydrazide derivatives were tested against phytopathogenic fungi, *Fusarium solani* and *Fusarium oxysporum*. Recent research has demonstrated that *Aspergillus fumigatus* poses a growing threat to the health of humans and livestock. It is one of the strongest allergens causing systemic fungal infections which are difficult to treat due to the pathogen’s high resistance to antifungal drugs [16,17].

The obtained compounds, in particular linear derivatives with fluorine-containing substituents, demonstrated satisfactory levels of biological activity. Further structural modifications could support the synthesis of compounds characterized by even higher antifungal potential. 

## 2. Results and Discussion

**Scheme 1:**
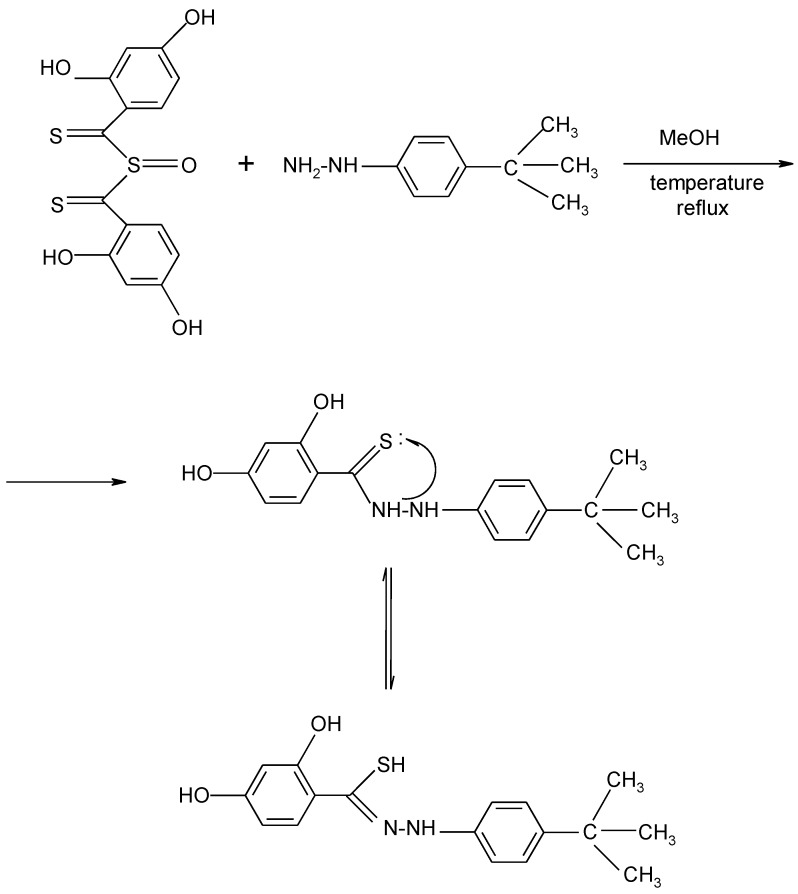
Possible synthetic pathway for the synthesis of compound **6**.

The synthesis of hydrazide derivatives demonstrated that the system tends to retain a linear structure regardless of the applied reagents (Scheme 1).

The structure of derivatives is presented in Table 1 and Table 2. In all cases, the results of elemental analysis were consistent with theoretical values. Compound purity was validated by RP-18 HPLC.

**Table 1:** Analytical data of compounds.

No	Molecular Formula	Yield [%]	Composition [%] calculated	Composition [%] found	Formula Weight	Molar Volume	m.p. °C
**1**	C_9_H_9_F_3_N_2_O_2_S	86	C (40.60) H (3.41) N (10.52)	C (40.5) H (3.42)	266.24	176.2 ± 3.0 cm^3^	115–117
**2**	C_9_H_12_N_2_O_2_S	73	C (50.92) H (5.70) N (13.20)	N (13.15)	212.26	159.8 ± 3.0 cm^3^	171–173
**3**	C_14_H_11_N_3_O_2_S	97	C (58.93) H (3.89) N (14.73)	--	285.32	191.1 ± 5.0 cm^3^	175
**4**	C_13_H_11_N_3_O_4_S	84	C (51.14) H (3.63) N (13.76)	C (51.1) H (3.61)	305.30	193.6 ± 3.0 cm^3^	166–167
**5**	C_13_H_10_F_2_N_2_O_2_S	79	C (52.70) H (3.40) N (9.45)	N (9.48)	296.29	190.2 ± 3.0 cm^3^	187–189
**6**	C_17_H_20_N_2_O_2_S	77	C (64.53) H (6.37) N (8.85)	C (64.45) H (6.32) N(10.03)	316.41	248.5 ± 3.0 cm^3^	104–107
**7**	C_15_H_16_N_2_O_2_S	75	C (62.48) H (5.59) N (9.71)	N (9.68)	288.36	214.3 ± 3.0 cm^3^	121–124
**8**	C_14_H_13_ClN_2_O_2_S	84	C (54.46) H (4.24) N (9.07)	--	308.78	210.0 ± 3.0 cm^3^	110–112
**9**	C_13_H_12_N_2_O_2_S	98	C (59.98) H (4.65) N (10.76)	--	260.31	181.8 ± 3.0 cm^3^	137–139
**10**	C_13_H_11_FN_2_O_2_S	82	C (56.10) H (3.98) N (10.07)	C (56.15) H (4.0) N (10.03)	278.30	186.0 ± 3.0 cm^3^	141–142

Infrared spectroscopy confirmed the presence of C-H stretching bands in the range of 3043 and 3000 cm^−1^, which are characteristic of aromatic structures. Weak overtone and combination bands were observed in the range of 1985 and 1650 cm^−1^ in all analyzed spectra. In-plane bending vibration bands C-H were reported in the range of 1270–1060 cm^−1^. The presence of absorption bands in the 1250–1020 cm^−1^ range, characteristic of thiocarbonyl compounds, was observed in all compounds.

Mass spectrometry analysis validated the structure of the examined derivatives. In all compounds, fragmentation peaks confirmed the structure of the analyzed bonds. Molecular ions were stable regardless of registration efficiency. In the spectra of compounds **1**, **3**, **5**, **8** and **9**, they were the main peaks of M^+^ ions.

**Table 2:** Structure of prepared compounds.

No	Structure	Log kw	S
**1**	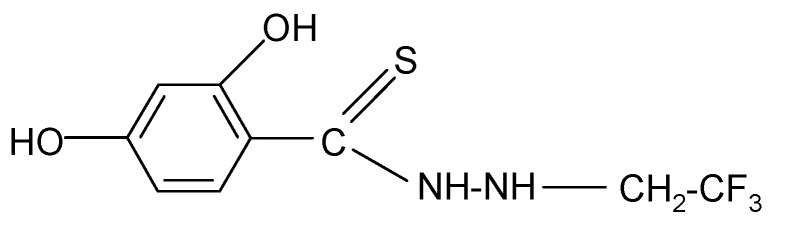	3.05	3.17
**2**	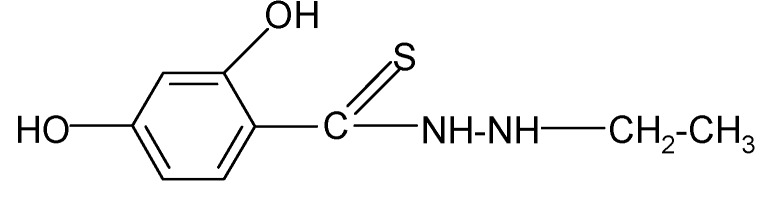	2.13	3.88
**3**	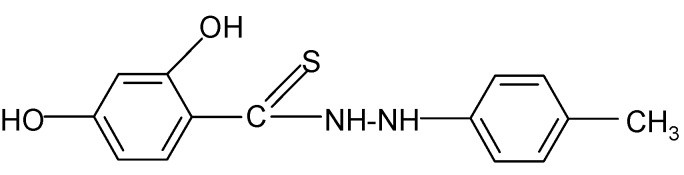	3.56	3.98
**4**	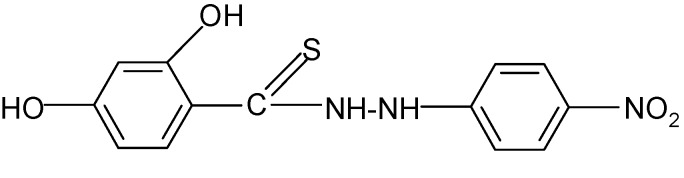	2.57	3.68
**5**	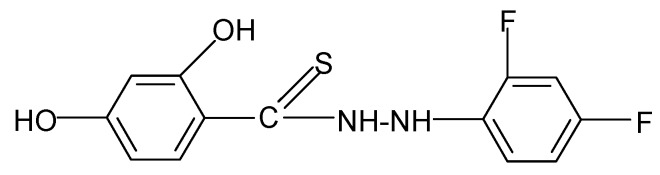	3.05	3.09
**6**	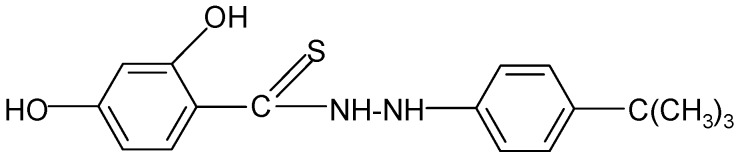	3.78	4.54
**7**	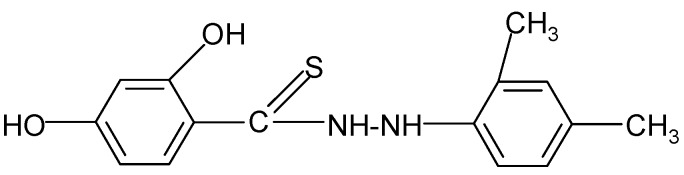	3.05	3.78
**8**	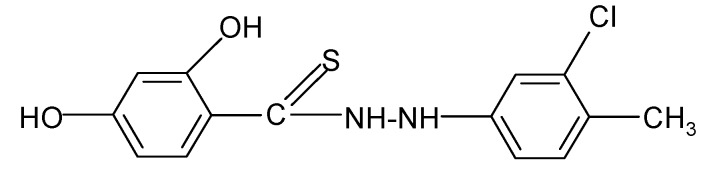	2.39	3.61
**9**	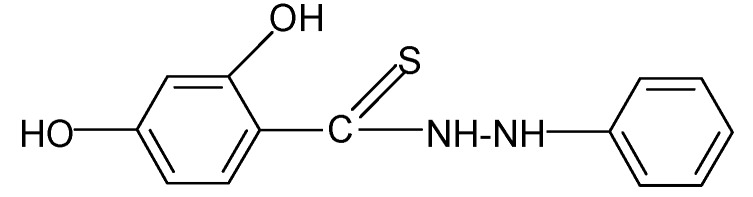	2.22	3.91
**10**	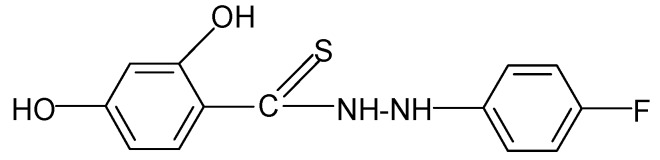	3.47	4.02

An analysis of the resulting ^1^H-NMR spectra supported the identification of three resonance lines in the aliphatic (4.18–4.41 ppm) and aromatic (7.15–8.48 ppm) range, as well as a singlet with two proton intensity in the range of 10.02–11.25 ppm, corresponding to the NH-NH group. The resonance signals of aromatic protons were observed in the form of doublets with coupling constants *J* = 8.5 Hz and 8.7 Hz.

**Figure 2:**
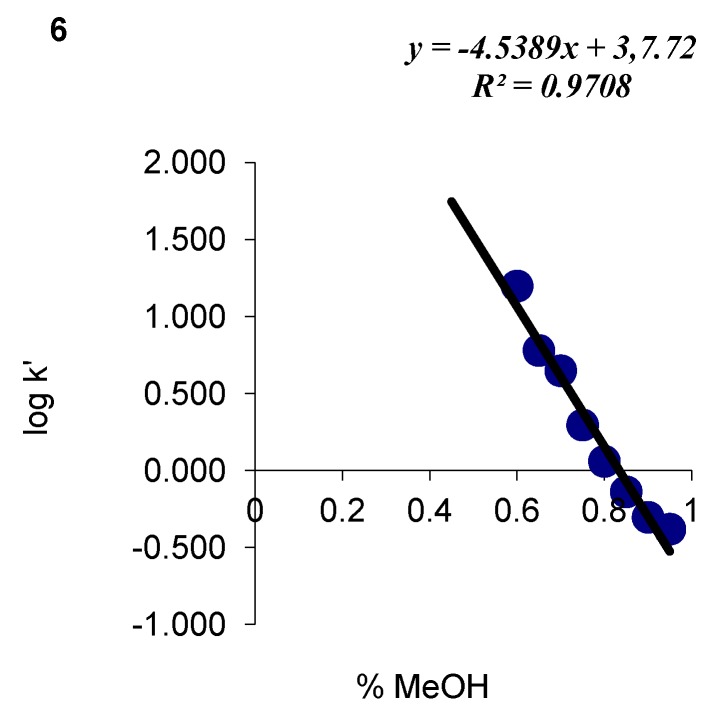
An experimental correlation between log k’ and the volume fraction of methanol mobile phase for compound 6.

**Figure 3:**
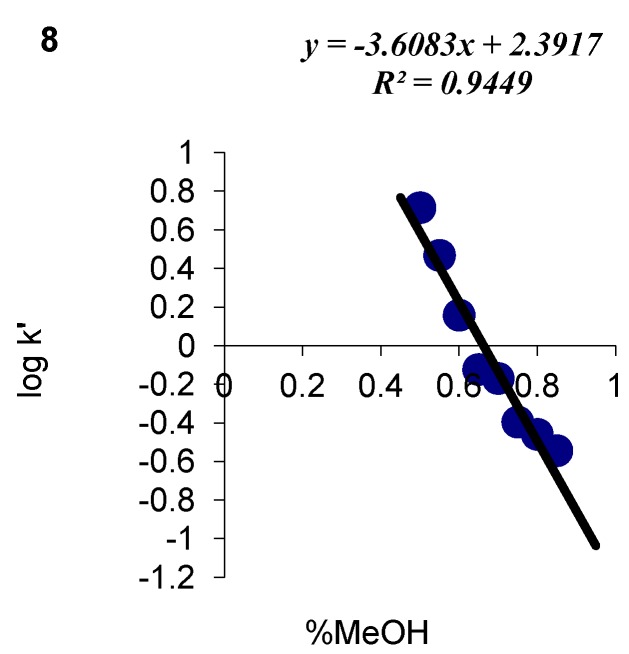
An experimental correlation between log k’ and the volume fraction of methanol mobile phase for compound **8**.

HPLC data for all analyzed compounds were used to determine the values of capacity factor log k’ and the volume fraction of the organic modifier (Figure 2 and Figure 3). The correlations between log k’ and φ were influenced not only by the properties of the chromatographic system, *i.e.*, the type of stationary phase and the concentration of substances in the mobile phase, but also the attributes and the structure of the examined substances. 

Methanol was used as an organic modifier in all experiments, therefore, the differences between the resulting curves can be attributed mostly to the physicochemical parameters of the analyzed compounds. The identified correlations were used to determine capacity factors in pure water log k_w_ by linear extrapolation. The volume fraction of methanol in the studied system reached φ = 0.45 − 0.95, therefore, log k_w_ could be determined from a sufficient number of points. 

The lipophilicity of the analyzed derivatives, presumably the key factor affecting cell transport and membrane penetration by molecules, was determined based on HPLC log kw parameters. Parameter log kw, the partition coefficient between water and the hydrophobic surface of the alkyl radical, seems to be the most robust descriptor of the studied compound. *S* is an equally important chromatographic parameter whose value is related to the elution force of the mobile phase modifier. It is determined mainly by the non-polar part of the compound as well as by the type and number of polar substituents in a molecule. The above parameter describes the rate of changes in substance retention at various concentrations of the applied organic modifier in the mobile phase. The linear correlation between log kW and S is described by the below formula:


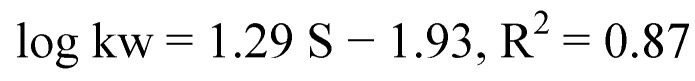


The produced linear correlation fulfils chromatographic requirements for determining hydrophobicity. The analyzed parameters can be applied interchangeably in further studies. 

The lipophilicity of the examined compounds was determined in the range of log kw 2.13−3.79. In the group of analyzed derivatives, the lowest lipophilicity values were noted for non-substituted 2,4-dihydroxy-*N*'-phenyl-benzenecarbothiohydrazide. An analysis of biological activity suggests that an absence of electron-donor and electron-acceptor substituents weakens or inhibits fungicidal activity. When compound 9 was applied at the concentration of 200 μg/mL, its fungicidal activity against *Fusarium solani* reached 61.1%, and against *Fusarium oxysporum*—Only 41.4%. The above compound showed no fungicidal action at concentrations below 200 μg/mL. An analysis of previous results [18,19] for differently substituted thioamides confirms that compounds of low lipophilicity are characterized by low levels of biological activity against pathogens. 

Derivatives with different substituents are marked by higher levels of biological activity (Table 3). They are also characterized by higher lipophilicity which enables a compound to move within a biological system, thus enhancing its biological activity. A higher capacity to migrate across membranes is required for a molecule to reach its target. 

Biological analyses of the *Fusarium solani* strain demonstrated that the fungicidal activity of three tested compounds (1, 6 and 10), applied at concentrations of 200 μg/mL, 100 μg/mL and 50 μg/mL, was similar to that of the popularly applied Amistar fungicide. Their lipophilicity varied in the range of 3.05–3.79 log kw. The presence of substituents: 2,2,2-Trifluoroethyl in the aliphatic system in compound 1, 4-*tert*-butyl in derivative 6, and 2-fluorophenyl in compound 10 enhanced the studied compounds’ lipophilicity and biological activity against the tested strains of pathogenic fungi.

The results of experiments investigating the fungicidal activity of hydrazide derivatives against *Aspergillus fumigatus* justify our previous work (Table 4). The investigated compounds are characterized by high fungicidal potential, which is a crucial consideration in the elimination of pathogens that quickly mutate, rapidly adapt to a new environment and exhibit high levels of toxicity, such as fungi of the genus *Aspergillus*. Azoxystrobin, a popular agent which is applied at low concentrations to fight fungal diseases in various crops, is not effective in 100%, which implies that it will cease to be a potent fungicide in several years’ time. 

**Table 3:** The fungicidal activity of the studied compounds against *Fusarium solani* and *Fusarium oxysporum*.

Compound number	*Fusarium solani*			*Fusarium oxysporum*
	200 μg/mL	100 μg/mL	50 μg/mL	200 μg/mL
**1**	3	3	3	3
**2**	3	2	1	1
**3**	3	3	2	3
**4**	3	3	1	1
**5**	3	2	2	3
**6**	3	3	3	3
**7**	3	3	2	3
**8**	3	3	2	1
**9**	2	1	0	1
**10**	3	3	3	1
Azoxystrobin	3	3	3	3

Effective inhibition of linear growth: [0]- 0–20%; [1]- 21–49.9%; [2]- 50–79.9%; [3]- 80–100%.

**Table 4:** The fungicidal activity of the studied compounds against *Aspergillus fumigatus*.

Compound number	*Aspergillus fumigatus*			
	200 μg/mL	100 μg/mL	50 μg/mL	25 μg/mL
**1**	3	3	2	2
**2**	3	3	3	3
**3**	2	2	2	0
**4**	2	1	1	0
**5**	3	1	1	1
**6**	3	3	3	3
**7**	3	2	2	2
**8**	2	2	0	0
**9**	1	1	0	0
**10**	3	3	3	3
Azoxystrobin	3	3	2	2

Effective inhibition of linear growth: [0]- 0–20%; [1]- 21–49.9%; [2]- 50–79.9%; [3]- 80–100%.

Our results confirmed high levels of fungicidal activity of compounds 6 and 10 at all applied concentrations. Mycelium growth was inhibited in 88–94% at all fungicide doses. The presence of tert-butyl and fluorine substituents at the para- position seems to guarantee high activity levels because molecules can easily penetrate biological barriers and quickly reach the molecular target. High levels of fungicidal activity were also reported for derivative **2** where the ethyl substituent is directly connected with a hydrazide group. The discussed compound is characterized by low lipophilicity, and further spectral and biological analyses are required to examine its structure and activity in greater detail. The results of our previous work [20] indicate that derivatives with chlorine- or fluorine-containing substituents with lipophilicity values in the range of 2.7–3.9 are most biologically active.

## 3. Experimental

### 3.1. General

Melting points were measured with a Boetius apparatus and are given uncorrected. The elemental analysis was performed using a Perkin-Elmer 2400 analyzer. The results of the analysis (C, H, N) were within the ±0.4% limit of the theoretical values. ^1^H-NMR spectra of solutions in DMSO-d_6_ solutions were analyzed in Varian 200 or Bruker 500 spectrometers with TMS as an internal standard. All chemical shifts were quoted in δ values (ppm). Infrared spectra were recorded using a Perkin-Elmer FT-IR 1725X spectrophotometer. The spectra were determined in the region of 600–4000 cm^−1^. MS spectra (EI 70 eV) were recorded using a 4000 QTRAP® Lc/MS/MS System, (AB SCIEX Germany Gmbh). 

### 3.2. Synthesis

(2,4-Dihydroxythiobenzoyl)hydrazides were synthesized from sulfinyl-bis-(2,4-dihydroxythiobenzoyl) (SHTB) and commercially available hydrazine derivatives. SHTB was obtained from 2,4-dihydroxythiobenzoic acid and SOCl_2_ according to a patent reaction [21]. The release of 2,4-dihydroxythiobenzoyl carbocations (C_6_H_3_(OH)_2_CS^+^) from SHTB as the result of solvolysis and thioamide bond formation was asynchronic, and it occurred without changes in carbon atom hybridization of the thiocarbonyl moiety.

*2,4-Dihydroxy-*N'*-(2,2,2-trifluoroethyl)benzene carbothiohydrazide* (**1**): 2,2,2-Trifluoroethylhydrazine solution (0.01 mol) and SHTB (0.005 mol) were placed in a round-bottom flask, and methanol (50 mL) was added. The synthesis was continued for 3 hours at a temperature of 65 °C by stirring the reagents. After the reaction, the hot mixture was filtered, and the filtrate was left to dry. It was twice rinsed with methanol (30 mL). ^1^H-NMR (DMSO-d_6_) δ (ppm): 3.67 (s, 3H, CH_3_), 10.08 (s, H, HOC-2), 11.34 (d, H, NH), 11.46 (s, H, HOC-4); EI-MS: *m/e* 267 [M+1]^+^ B=100%; IR (cm^−1^): 3330 ν s,as OH, NH; 2975, 2926, 2889 ν s,as CH, ν C-Ar H; 1450 ν -NHC(S=)-; 1379 δ as, C-H; 1274 ν C…N; 1047 ν C=S.

*2,4-Dihydroxy-*N'*-ethylbenzene carbothiohydrazide* (**2**): Ethyl hydrazine (0.01 mol) and SHTB (0.005 mol) were placed in a round-bottom flask and methanol (50 mL) was added. The synthesis was continued for 3.5 hours at a temperature of 95 °C by stirring the reagents. After the reaction, the hot mixture was filtered, and the filtrate was left to dry. The dried product was dissolved in heated 5:1 methanol-water mixture (50 mL). The final product was filtered and twice rinsed with methanol (25 mL). ^1^H-NMR DMSO-d_6_) δ (ppm): 4.25 (m, 5H, CH), 9.90 (s, H, HOC-2), 11.15 (d, H, NH), 11.32 (s, H, HOC-4); EI-MS: *m/e* 208 [(HO)_2_C_6_H_3_-C_3_H_3_N_2_S] B=100%; IR (cm^−1^): 3337 ν s,as OH, NH; 2974, 2927, 2889 ν s,as CH, 1631, 1527, 1449 ν C=C; 1380 δ as, C-H; 1047 ν C=S; 880 δ C_ar_H. 

N'*-(4-Cyanophenyl)-2,4-dihydroxybenzene carbothiohydrazide* (**3**): 4-Cyanophenylhydrazine hydro-chloride (0.01 mol) and SHTB (0.005 mol) were placed in a round-bottom flask and methanol (50 mL) was added. The synthesis was continued for 4 hours at a temperature of 95 °C by stirring the reagents. After the reaction, the hot mixture was filtered, and the filtrate was left to dry. The end product was twice rinsed with methanol (20 mL). ^1^H-NMR DMSO-d_6_) δ (ppm): 7.62–7.81 (m, 4H, arom.), 10.05 (s, H, HOC-2), 10.74 (d wide, H, NH), 11.46 (s, H, HOC-4); EI-MS: *m/e* 284 [M-1]^+^ B=100%; IR (cm^−1^): 3330 ν s,as OH, NH; 2974, 2926, 2889 ν s,as CH, 1544 ν C=C; 1445 ν ‑NHC(=S)-; 1328 ν C=C; 1047 ν C=S; 975 δ C_ar_H (in plane); 880, 803, 752, 738 δ C_ar_H (out of plane).

*2,4-Dihydroxy-*N'*-(4-nitrophenyl)benzene carbothiohydrazide* (**4**): 4-Nitrophenylhydrazine hydro-chloride (0.01 mol) and SHTB (0.005 mol) were placed in a round-bottom flask and methanol (50 mL) was added. The synthesis was continued for 3.5 hours at a temperature of 95 °C by stirring the reagents. After the reaction, the hot mixture was filtered, and the filtrate was left to dry. The end product was dissolved in 3:1 methanol-water mixture, it was filtered and twice rinsed with methanol (20 mL). ^1^H-NMR DMSO-d_6_) δ (ppm): 7.42–7.60 (m, 4H, arom.), 10.21 (s, H, HOC-2), 10.98 (d wide, H, NH), 11.78 (s, H, HOC-4); EI-MS: *m/e* 136 [NO_2_C_6_H_4_NH]^+^ B=100%; IR (cm^−1^): 3061 ν C_ar_H (in plane); 2889, 2850, 2760 ν s,as CH, 1641, 1586 ν C=C; 1513 ν -NHC(=S)-; 1351 ν NO_2_; 1027 ν C=S; 963 δ C_ar_H (in plane).

N'*-(2,4-Difluorophenyl)-2,4-dihydroxybenzene carbothiohydrazide* (**5**): 2,4-Difluorophenylhydrazine hydrochloride (0.01 mol) and SHTB (0.005 mol) were placed in a round-bottom flask and methanol (50 mL) was added. The synthesis was continued for 3.5 hours at a temperature of 110 °C by stirring the reagents. After the reaction, the hot mixture was filtered, and the filtrate was left to dry. The dried product was dissolved in heated 3:1 methanol-water mixture (40 mL) with the addition of 2 M HCl, and it was heated for 2 hours at a temperature of 50 °C. The final product was filtered and twice rinsed with methanol (20 mL). ^1^H-NMR DMSO-d_6_) δ (ppm): 7.01–7.12 (m, wide, shift, 3H, arom.), 9.88 (s, H, HOC-2), 10.03 (d, H, NH), 11.65 (s, H, HOC-4); EI-MS: *m/e* 298 [M+2]^+^ B=100%; IR (cm^−1^): 3327, 3227 ν s,as OH, NH; 3094 ν C_ar_H (in plane); 2976, 2927, 2889, 2851 ν s,as CH, 1619, 1588 ν C=C; 1502 ν -NHC(=S)-; 1342 ν C-F; 1046 ν C=S; 981 δ C_ar_H (flat); 879, 847 δ C_ar_H (out of plane).

N'*-(4-tert-Butylphenyl)-2,4-dihydroxybenzene carbothiohydrazide* (**6**): 4-tert-Butylphenylhydrazine hydrochloride (0.01 mol) and SHTB (0.005 mol) were placed in a round-bottom flask, and 50 mL methanol was added. The synthesis was continued for 3 hours at a temperature of 65 °C by stirring the reagents. After the reaction, the hot mixture was filtered, and the filtrate was left to dry. The final product was twice rinsed with methanol (30 mL). ^1^H-NMR DMSO-d_6_) δ (ppm): 1.87 (s, 3H, CH_3_), 2.03 (s, 3H, CH_3_), 2.06 (s, 3H, CH_3_), 7.14–7.42 (m, 4H, arom.), 10.09 (s, H, HOC-2), 10.12 (d, H, NH), 11.88 (s, H, HOC-4); EI-MS: *m/e* 185 [(HO)_2_C_6_H_3_C(=S)NHNH +2] B=41,07%; IR (cm^−1^): 2922, 2864 ν s,as CH; 1631, 1587 ν C=C; 1513 ν -NHC(=S)-; 1452, 1443 δ CH_3_; 1032 ν C=S.

N'*-[(2,4-Dimethyl)phenyl]-2,4-dihydroxybenzene carbothiohydrazide* (**7**): (2,4-Dimethyl)phenyl hydrazine sulfate (0.01 mol) and SHTB (0.005 mol) were placed in a round-bottom flask and methanol (50 mL) was added. The synthesis was continued for 2.5 hours at a temperature of 65 °C by stirring the reagents. After the reaction, the hot mixture was filtered, and the filtrate was left to dry. The final product was twice rinsed with methanol (30 mL). ^1^H-NMR DMSO-d_6_) δ (ppm): 2.19 (d, 6H, CH_3_), 3.67 (d, 4H, CH_2_), 7.41–7.94 (m, 3H, arom.), 10.14 (s, H, HOC-2), 11.06 (d, H, NH), 11.67 (s, H, HOC-4); EI-MS: *m/e* 284 [M-4]^+^ B=100%; IR (cm^−1^): 3635, 3343, 3148 ν s,as OH, NH; 2975, 2927, 2884, 2851 ν s,as CH; 1524, ν C=C; 1450 δ CH_3_; 1047 ν C=S; 961 δ C_ar_H (in plane); 880, 835, 793, 726 δ C_ar_H (out of plane).

N'*-(3-Chloro-p-tolylphenyl)-2,4-dihydroxybenzene carbothiohydrazide* (**8**): 3-Chloro-*p*-tolylphenyl hydrazine hydrochloride (0.01 mol) and SHTB (0.005 mol) were placed in a round-bottom flask and methanol (50 mL) was added. The synthesis was continued for 3 hours at a temperature of 65 °C by stirring the reagents. After the reaction, the hot mixture was filtered, and the filtrate was left to dry. The final product was twice rinsed with methanol (20 mL). ^1^H-NMR DMSO-d_6_) δ (ppm): 1.64 (s, 3H, CH_3_), 7.16–7.44 (m, 3H, arom.), 10.20 (s, H, HOC-2), 10.35 (d, H, NH), 11.84 (s, H, HOC-4); EI-MS: *m/e* 309 [M]^+^ B=100%; IR (cm^−1^): 2929, 2889, 2851, 2838 ν s,as CH; 1631, 1602, ν C=C; 1526 ν -NHC(S=)-; 1470 ν C=C; 1450 δ CH_3_; 1047 ν C=S; 842, 684 δ C-Cl; 879, 858, 795, 758, 728 δ C_ar_H (out of plane).

N'*-Phenyl-2,4-dihydroxybenzene carbothiohydrazide* (**9**): Phenylhydrazine hydrochloride (0.01 mol) and SHTB (0.005 mol) were placed in a round-bottom flask and methanol (50 mL) was added. The synthesis was continued for 4 hours at a temperature of 85 °C by stirring the reagents. After the reaction, the hot mixture was filtered, and the filtrate was left to dry. The dried product was dissolved in heated 5:1 methanol-water mixture, filtered and twice rinsed with methanol (20 mL). ^1^H-NMR DMSO-d_6_) δ (ppm): 7.16–7.36 (m, 4H, arom.), 10.03 (d, H, NH), 10.09 (s, H, HOC-2), 11.77 (s, H, HOC-4); EI-MS: *m/e* 259 [M-1]^+^ B=100%; IR (cm^−1^): 2974, 2927, 2884 ν s,as CH; 1524 ν ‑NHC(S=)-; 1450 ν C=C; 1143 δ C_Ar_ H; 1047 ν C=S; 880, 801, 779, 735, 718 δ C_ar_H (out of plane).

N'*-(2-Fluorophenyl)-2,4-dihydroxybenzene carbo thiohydrazide* (**10**): 2-Fluorophenylhydrazine monohydrochloride (0.01 mol) and SHTB (0.0025 mol) were placed in a flask and methanol (25 mL) was added. The mixture was heated at 75 °C for 1.5 hours, and SHTB (0.0025 mol) and methanol (25 mL) were added. The synthesis was continued for 2.5 hours at a temperature of 85 °C by stirring the reagents. After the reaction, the hot mixture was filtered, and the filtrate was left to dry. The final product was twice rinsed with methanol (40 mL). ^1^H-NMR DMSO-d_6_) δ (ppm): 7.15–7.40 (m, 4H, arom.), 9.96 (s, H, HOC-2), 9.98 (d, H, NH), 11.00 (s, H, HOC-4); EI-MS: *m/e*149 [C_6_H_5_C(=S)N=N-]^+^ B=100%; IR (cm^−1^): 3343, 3334 ν s,as OH, NH; 2974, 29276, 2889, 2853 ν s,as CH; 1513 ν ‑NHC(S=)-; 1460, 1450 ν C=C; 1089 ν C-F; 1048 ν C=S; 880, 803 δ C_ar_H (out of plane).

### 3.3. Chromatography (HPLC)

The purity of the compounds was checked using a liquid chromatograph (Knauer) equipped with a dual pump, a 20 μL sample injection valve and a UV-VIS detector (320 nm). A Hypersil BDS C_18_ column (5 μm, 150 × 4.6 mm) was used as the stationary phase. The mobile phase consisted of various methanol concentrations with 10 mL acetate buffer (pH = 4) as the aqueous phase. The flow rate was 0.5 mL/min. at room temperature. Column dead time was determined by the injecting a small amount of acetone dissolved in water. To calculate solute lipophilicity, capacity factors (k) have to be determined for different concentrations of the organic modifier. Log kw and S can be calculated by on the assumption that log k and φ are bound by a linear correlation, as, expressed by the Soczewiński-Wachtmeister equation:


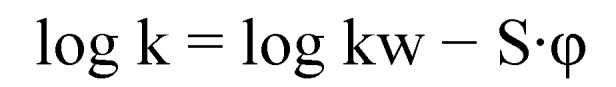


where log kw = the logarithm of the theoretical value of k for pure water as the mobile phase (widely used as a measure of lipophilicity in QSAR). S = the difference between log k for pure water and the pure modifier (indicative of the retention mechanism).

### 3.4. Biological Assays

*In vitro* tests of fungicidal activity against *Fusarium solani* and *Fusarium oxysporum* and *Aspergillus fumiagatus* fungi were performed. The fungi were isolated from plant material and identified according to standard mycological procedures. Pathogens were identified based on a macroscopic analysis of the morphological properties of the mycelium and conidial spores with the use of diagnostic keys [22,23,24]. 

A standard fungicide, Amistar 250 SC (active ingredient: azoxystrobin), was used. The tests were carried out in line with the laboratory procedure for evaluating fungicidal activity (procedure SPR/FA_2_/11). The objective of the analysis was to estimate the effect of various concentrations of the applied compound on fungal colony growth. The fungicide was added in the form of a solution or suspension to sterile and cooled (45 °C) PDA medium (3.9 g DDA medium, Difco, with 0.2 g agar/100 mL distilled water) to produce fungicide concentrations of 200 μg/L, 100 μg/ L and 50 μg/ L in the medium. In tests investigating the compounds’ fungicidal activity against *Aspergillus fumiagatus*, the lowest concentration of diluted substance was 25 μg/ L. The analyzed compounds were dissolved in DMSO. The fungicide was dissolved in water. 

Liquid medium containing the studied substances was poured into Petri dishes with a diameter of 9 cm. After medium solidification, the center of the dish was inoculated with 5 mm overgrown mycelium of the investigated species. The dishes containing strains of the genus *Fusarium* were incubated at 21 °C for 7 days. *Aspergillus fumigatus* strains were incubated at 37 °C for 7 days. 

Plates containing only DMSO without the studied compound served as control. After incubation, average diameters of the cultured fungal colonies were measured. Abbot’s formula was used to calculate the compounds’ or the standard’s effectiveness in inhibiting the linear growth of mycelia at various concentrations:


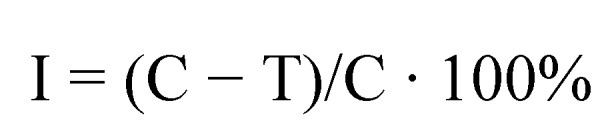


I - percentage of mycelial growth inhibition,

C - colony growth zone in control treatment (mm)

T - colony growth zone in experimental treatment (mm)

The following evaluation criteria were applied in Table 5:

**Table 5:** Compound’s effectiveness in inhibiting the linear growth of mycelia in%.

Effective inhibition of linear growth in %	Evaluation of fungicidal activity	
	Degree on scale	Activity
Above 80	3	satisfactory
50–79.9	2	average
20–49.9	1	weak
Below 20	0	no activity

The experiments was performed with two replicates per treatment.

## 4. Conclusions

In view of the biological activity of the studied hydrazides, as well as the results of our previous studies, we believe that further work is needed to synthesize new derivatives with different structure.

The parameters determined using liquid chromatography can be applied interchangeably to identify the lipophilicity of synthesized compounds.

The produced compounds demonstrate satisfactory levels of biological activity against the isolated fungal strains of the genus *Fusarium*. Further research is required to investigate the activity of the newly synthesized derivatives against other strains of fungal pathogens infecting crop plants, such as *F. culmorum* and *F. oxysporum*. Those efforts will contribute to the prevention of cereal infections by fungal pathogens of the genus *Fusarium* and grain contamination with *Fusarium* mycotoxins.

The analyzed derivatives were characterized by satisfactory fungicidal activity against fungi of the genus *Aspergillus*, pathogens that pose a serious threat for both humans and crops. Compounds exhibiting lower levels of fungicidal activity will be tested to determine their effectiveness against yeast-like fungi and dermatophytes which have different structure and properties.

## References

[1] Danish I.A., Prasad K.J. (2004). Syntheses and characterization of *N,N'*-biscarbazolyl azine and *N,N'*- hydrazine derivatives and their antimicrobial studies. Acta Pharm..

[2] Suthakaran R., Kavimani S., Venkappayya D., Jayasree P., Deepthi V., Tehseen F., Suganthi K. (2008). Microwave assisted rapid synthesis and anti-microbial activity of 4-oxothiazolidine derivatives. Rasayan J. Chem..

[3] Juszczak M., Matysiak J., Brzana W., Niewiadomy A., Rzeski W. (2008). Evaluation of the antiproliferative activity of 2-(monohalogenophenyloamino)-5-(2,4-dihydroxyphenyl)-1,3,4-thiadiazoles. Arzneimittelforschung.

[4] Niewiadomy A., Krajewska-Kułak E., Łukaszuk C., Matysiak J., Kostecka M. (2005). In vitro antifungal activity of * N*-3-(1,2,4-dithiazole-5-thione)-beta-resorcylcarbothioamide. Rocz. Akad. Med. Białymst..

[5] Bredihhin A., Groth U.M., Mäeorg U. (2007). Formation and use of a nitrogen dianion for selective hydrazine alkylation provides a fast and easy access to substituted hydrazines, which are widely used as drugs, pesticides, and precursors for a variety of compounds in organic synthesis. Org. Lett..

[6] Tšubrik O., Sillard R., Mäeorg U. (2006). Excellent regioselectivity is observed in the addition of diverse organometallic nucleophiles to unsymmetrical azo compounds. Primary/secondary/tertiary alkyl, aryl and heteroaryl substituents were introduced this way in high yields. Synthesis.

[7] Bredihhin A., Mäeorg U. (2007). . Alkylation of hydrazine using a polyanion strategy provides a fast and convenient access to multialkylated hydrazine derivatives. Scope and limitations of the new method are also investigated. Org. Lett..

[8] Manivel P., Mohana Roopan S., Sathish Kumar R., Nawazkhan F. (2009). Synthesis of 3 substituted isoquinolin-1-yl-2-(cycloalk-2-enyldiene) hydrazines and their antimicrobial properties. J. Chil. Chem. Soc..

[9] Sherine N.K. (2005). Synthesis and biological activity of novel amino acid-(N'-Benzoyl) hydrazide and amino Acid-(N'-nicotinoyl)hydrazide derivatives. Molecules.

[10] Sechi M., Azeena U., Delussu M.P., Dallochio R., Dessi A., Cosseddu A., Pala N., Neamati N. (2008). Design and synthesis of bis-amide and hydrazide containing derivatives of malonic acid as potential HIV-1 integrase inhibitors. Molecules.

[11] Mohareb R.M., Mohamed A.A. (2010). The reaction of cyanoacetylhydrazine with ω-bromo-(4-methyl)acetophenone: Synthesis of heterocyclic derivatives with antitumor activity. Molecules.

[12] Al-Abdullah E.S. (2011). Synthesis and anticancer activity of some novel tetralin-6-yl-pyrazoline, 2-thioxopyrimidine, 2-oxopyridine,2-thioxo-pyridine and 2-iminopyridinederivative. Molecules.

[13] Umelsalma S., Sudhandiran G. (2011). Ellagic acid prevents rat colon carcinogenesis induced by 1,2- dimethyl hydrazine through inhibition of AKT-phosphoinosotide-3-kinase pathway. Eur. J. Pharmacol..

[14] Wandall H.H., Tarp M.A., Guo Z., Boons G.-J. (2008). Therapeutic cancer vaccines: Clinical trials and applications. In Carbohydrate-Based Vaccines and Immunotherapies.

[15] Kaur R., Singh A., Budhlakoti P., Singh A., Sanwal R. (2010). Synthesis, characterization and antifungal activity of certain(*E*)-1-(1-(substitutedphenyl) ethylidene)-2-(6-methylbenzo [d] thiazol-2-yl) hydrazine analogues. Int. J. Pharm. Biol. Arch..

[16] Dagenais R.T., Keller N.P. (2009). Pathogenesis of *Aspergillus fumigatus* in invasive aspergillosis. Clin. Microbiol. Rev..

[17] Wild C.P. (2007). Aflatoxin exposure in developing countries: The critical interface of agriculture and health. Food Nutr. Bull..

[18] Kostecka M., Niewiadomy A., Czeczko R. (2005). Evaluation of *N*-substituted 2,4-dihydroxyphenylthioamide fungicide lipophilicity using the chromatographic techniques HPLC and HPTLC. Chromatographia.

[19] Legocki J., Matysiak J., Niewiadomy A., Kostecka M. (2003). Synthesis and fungistatic activity of new groups of 2,4-dihydroxythiobenzoyl derivatives against phytopathogenic fungi. J. Agric. Food Chem..

[20] Kostecka M. (2010). Search for an efficient compound with antifungal properties inhibiting Fusarium genus fungi. Ecol. Chem. Eng. A.

[21] Niewiadomy A., Matysiak J., Mącik-Niewiadomy G. Nowe tioamidy, produkt poÅśredni do otrzymywania nowych tioamidów.

[22] Gerlach W., Nirenberg H. (1982). The Genus Fusarium—A Pictorial Atlas. In Mitteilungen aus der Biologischen Bundesanstalt fur Land- und Forstwirtschaft (in Germany).

[23] Muthomi J.W., Schutze A., Dehne H.W., Mutitu E.W., Oerke E.C. (2000). Characterization of *Fusarium culmorum* isolates by mycotoxin production and aggressiveness to winter wheat. J. Plant Dis. Prot..

[24] Nelson P.E., Toussoun T.A., Marasas W.F.O. (1983). Fusarium Species-An Illustrated Manual for Identification.

